# Epidemiological, virological, and pathogenic insights into Nairobi sheep disease virus infection in sheep and goats in China

**DOI:** 10.1128/jvi.00006-25

**Published:** 2025-05-21

**Authors:** Xin-Yan Yao, Meng-Hang Wang, Xue-Lian Zhang, Xu Zhang, De-Xin Liang, Shao-Han Li, Chun-Yang Lian, Zhi-Hang Lv, Chang-You Xia, Ming-fa Yang, Jian-Wei Shao, Xin Yin

**Affiliations:** 1 School of Animal Science and Technology, Foshan University 47868, Foshan, China; 2 State Key Laboratory for Animal Disease Control and Prevention, Harbin Veterinary Research Institute of Chinese Academy of Agricultural Sciences 687216 , Harbin, China; University Medical Center Freiburg, Freiburg, Germany

**Keywords:** Nairobi sheep disease virus, identification, epidemiology, virus isolation, pathogenic characterization, sheep and goats

## Abstract

Nairobi sheep disease virus (NSDV), a tick-borne pathogen in the *Orthonairovirus* genus of the *Nairoviridae* family, causes Nairobi sheep disease (NSD), which has mortality rates over 90% in sheep and goats. Historically, NSD outbreaks in East Africa and South Asia have led to considerable economic losses. Although NSDV genomic fragments have been detected in ticks in China, there has been a notable absence of reported infections in local sheep and goats. In this study, we report the first identification of NSDV infection in sheep and goats in China. Through meta-transcriptomics sequencing, NSDV was found in infected spleen tissue with high viral abundance. Epidemiological investigations indicated a 4.1% positive rate for NSDV RNA and a 29.9% seroprevalence rate, confirming the presence of NSDV infection in these herds. Sequence analyses showed that the identified NSDV clustered with those previously found in ticks in China but were distinct from Africa or India strains. Furthermore, these viruses could be categorized into different sub-clades, suggesting the genetic diversity of NSDV in China. Remarkably, NSDV was isolated from infected sheep for the first time in China, and the isolated virus could cause severe clinical signs, leading to sheep mortality. In summary, this study represents the initial identification of NSDV in vertebrate hosts in China and highlights the importance of NSDV surveillance to prevent outbreaks of NSD within the country.

**IMPORTANCE:**

As a significant tick-borne virus, Nairobi sheep disease virus (NSDV) has caused devastating disease known as Nairobi sheep disease (NSD) in small ruminants in East Africa and South Asia. Although NSDV genomic fragments have been detected in ticks within China, there has been little information on its infection in local sheep or goats. This study confirms, for the first time, the infection of NSDV among these animals in China. Additionally, the successful isolation of NSDV from infected sheep highlights its capacity to replicate in various cell lines, including those of human origin, thereby underscoring its broad host range and potential for cross-species transmission. Notably, animal experiments demonstrated that the isolated virus strain could cause severe clinical signs in sheep. These findings provide the first evidence for the presence of NSDV infection among sheep and goats in China, underscoring the critical need for NSDV surveillance to prevent outbreaks of NSD within the country.

## INTRODUCTION

The genus *Orthonairovirus*, which is part of the family *Nairoviridae*, currently includes 15 official species primarily transmitted by ticks (1). Members of the genus *Orthonairovirus* have been classified into nine genogroups based on phylogenetic relationships, with the Nairobi sheep disease (NSD) genogroup being the most relevant for public and veterinary significance (2). The NSD genogroup comprises Crimean-Congo hemorrhagic fever virus (CCHFV), Nairobi sheep disease virus (NSDV), Hazara virus, Dugbe virus, Kupe virus, and Tofla virus (2). Among these, CCHFV infection can lead to fatal hemorrhagic fever in humans, while NSDV can cause fatal NSD in sheep and goats (1). The genome of orthonairoviruses is characterized by negative-sense single-stranded RNA consisting of three segments: the large (L) segment RNA encodes RNA-dependent RNA polymerase (RdRp), the medium (M) segment RNA encodes the glycoprotein precursor (GPC), and the small (S) segment RNA encodes the structural nucleoprotein (N) (1).

The NSD caused by NSDV infections is recognized as one of the most pathogenic diseases affecting sheep and goats and has been listed as a notifiable animal disease by the World Organization for Animal Health (WOAH) for many years (3). NSD was initially observed in Nairobi, Kenya, in 1910, and its causative agent NSDV was firstly determined in 1917 (4). Historically, NSDV was endemic to East African countries, including Uganda, Ethiopia, Somalia, Tanzania, and Rwanda (5–9). More recently, the occurrence of Ganjam virus, the Asian variant of NSDV, has been reported in India and Sri Lanka (10–12), indicating the wide geographical distribution of NSDV. NSDV infection in sheep and goats manifests as acute hemorrhagic gastroenteritis, leading to mortality rates as high as 90% and causing significant economic losses for lamb producers (10). Notably, serological evidence has confirmed NSDV infection in humans in India (13–15) and Kenya (16), suggesting its zoonotic potential.

In China, there is a significant presence of small-scale sheep and goat farms that maintain herds ranging from dozens to hundreds of animals. These small farms contribute to 80% of ovine meat production and play crucial roles in supporting the ovine meat industry (17). However, the occurrence of both known and unknown causes of emerging infectious diseases often leads to substantial morbidity and mortality rates among sheep and goats. This results in severe economic losses for rural farmers and presents significant challenges to the sustainable and healthy development of the sheep and goat industry. Therefore, conducting comprehensive surveys and implementing effective disease monitoring measures are essential steps to safeguard socio-economic development and ensure animal welfare.

As with other orthonairoviruses, NSDV is predominantly transmitted by ticks. NSDV genomic fragments were initially discovered in *Haemaphysalis longicornis* ticks in northeastern China in 2013 (18). Emerging studies have proven that NSDV could be detected in *H. longicornis* ticks across various regions of China (19–23), suggesting its widespread distribution in the country. Nonetheless, there have been no reports of NSDV infections in domestic animals or humans in China. In this study, for the first time, NSDV was identified in the spleen of sheep and goats collected from rural areas in Henan Province, China, using unbiased high-throughput meta-transcriptomic sequencing and virus isolation. The complete genome sequence of this novel NSDV strain MZ-18 was determined, and the NSDV clinical strain was then isolated and characterized. This study provides the first evidence of NSDV infection in sheep and goats in China and emphasizes the importance of NSDV surveillance to prevent outbreaks of NSD within the country.

## RESULTS

### Identification and abundance estimation of NSDV in the spleen of sheep and goats

In a pilot viral agent discovery project in sheep and goats, 90 spleen tissues were collected. Initially, RNA samples extracted from 30 individual spleen tissue of sheep and goats were mixed into one pool, and meta-transcriptome sequencing generated a total of 22,672,367 paired reads. Through *de novo* assembly and comparison against the nr database, 27 contigs ranging from 263 to 1,585 nt in length were annotated as NSDV. Among them, 14, 12, and 1 contigs were found to be derived from the L, M, and S segments of NSDV, respectively. To confirm the presence of NSDV in sheep and goats, RNA samples prior to pooling were subjected to nested PCR that targeted the L segment of NSDV, and one sample (MZ-18) tested positive for NSDV. The complete genome sequences of NSDV strain MZ-18 were determined by reverse transcription-PCR (RT-PCR) to fill the gaps and rapid amplification of cDNA ends (RACE) to obtain the genomic terminus.

To estimate the viral abundance within individual samples, total RNA of MZ-18 was further subjected to meta-transcriptome sequencing. In the library of MZ-18, three contigs ranging from 2,724–1,2014 nt in length were annotated as the genome of NSDV. Strikingly, the nearly complete L, M, and S segments were assembled directly. In addition, 7,395 non-rRNA reads were mapped to the determined complete genome sequence of NSDV strain MZ-18, with a >98.4% genome coverage and a mean depth of >38.1× (Fig. S1). Moreover, the relative abundance of NSDV strain MZ-18 was 101, 31, and 32 reads per million in the library (RPM) for L, M, and S segments, respectively (Fig. S1).

### Genetic characterization of NSDV strain MZ-18

The complete sequence of L, M, and S segments of NSDV strain MZ-18 contains 12,080, 5,083, and 1,601 nucleotides, respectively. The L segment RNA encodes a putative RdRp with 3,991 aa, while the M and S segment RNAs encode the putative GPC (1,628 aa) and N protein (482 aa). The 3′-NCR of L, M, and S segments was 42 bp, 53 bp, and 51 bp, respectively, while the 5′-NCR of L, M, and S segments was 65 bp, 146 bp, and 104 bp, respectively (Fig. S1). All three segments shared the genomic terminus 5′-UCUCAAAGA and 3′-UCUUUGAGA, which were the same as those of other orthonairoviruses (1).

The RdRp of NSDV strain MZ-18 contains several presumed regions and domains, including an ovarian tumor-like protease domain, a polymerase module (pre-motif A, motif A–E), and region I, region II, and region IV, which were conserved among the NSD genogroup members in the genus of *Orthonairovirus* (Fig. S2). The GPC of NSDV strain MZ-18 is cleaved into mature Gn and Gc by subtilisin/kexin-isozyme-1 protease at the predicted cleavage sites RRLM_453_ and RKLL_976_ (Fig. S1). The Gn protein of NSDV strain MZ-18 is predicted to contain two *N*-glycosylation sites, two transmembrane domains, and a pair of conserved zinc-finger domains, which have also been found in other NSD genogroup viruses (Fig. S3). Similarly, the Gc protein of NSDV strain MZ-18 contains three *N*-glycosylation sites, a C-terminus localized transmembrane, and three fusion loop regions that are conserved in NSD genogroup viruses (Fig. S4). Additionally, the head domains of NSDV N protein were highly conserved with other NSD genogroup viruses, while the stalk domain exhibits apparent high flexibility among the NSD genogroup viruses (Fig. S5). Notably, the Gn and Gc proteins display high conservation between NSDV strains identified from mammals and ticks (Fig. S6).

### Sequence comparison and phylogenetic analysis of NSDV strain MZ-18

Sequence comparison analysis showed that the coding region of L, M, and S segments of NSDV strain MZ-18 shared 88.7%–98.1%, 74.8%–96.5%, and 88.2%–95.9% nucleotide sequence identity with other known NSDV strains. With amino acid sequences, the RdRp, GPC, and N proteins of NSDV strain MZ-18 exhibited 96.5%–99.0%, 81.8%–97.8%, and 97.3%–99.0% identity with others (Table 1). The coding region of L segment and M segment RNA, as well as their encoded RdRp protein and the GPC protein of NSDV strain MZ-18, displayed the highest nucleotide and amino acid sequence identity with the NSDV strains TIGMIC_1 and Hubei, respectively, which were identified from *H. longicornis* ticks in China (19, 20). The nucleoprotein gene of MZ-18 shared the highest nucleotide sequence identity with the NSDV strain Jilin that was identified in *H. longicornis* in Jilin Province of China, whereas the N protein displayed the highest amino acid sequence identity with NSDV strain nlb-dd-tick that was identified from *H. longicornis* in Liaoning Province, China (Table 1).

**TABLE 1:** Identity of coding region sequence and amino acid sequence of NSDV strain MZ-18 with other NSDV strains^*a*^

Virus strain	nt sequence identity (%)			aa sequence identity (%)		
	L	M	S	RdRp	GPC	N
SZWH2/tick/China	95.9	95.2	94.2	98.2	97.3	98.8
Hubei/tick/China	95.8	96.5	94.8	97.7	97.8	98.1
Jilin/tick/China	95.3	94.4	95.9	97.8	96.9	98.1
nlb-dd-tick/tick/China	96.0	95.4	94.9	98.4	97.5	99.0
TIGMIC_1/tick/China	98.1	–^*b*^	–	99.0	–	–
708/tick/Kenya	88.9	87.0	88.2	96.5	92.9	96.3
Ganjam G619/tick/India	88.7	74.8	88.3	96.5	81.8	97.3
779/human/India	88.7	74.8	88.3	96.5	81.8	97.3

^*a*^ Sequences were aligned using the ClustalW method, and sequence identity was calculated using MegAlign. The GenBank accession numbers for the L, M, and S segments of NSDV strains used in this study are as follows: SZWH2 (MZ965002, MZ965003, MZ965004); Hubei (MH791449, MH791450, MH791451); Jilin (NC034387, NC034391, NC034386); nlb-dd-tick (OQ581155, OQ581154, OQ581153); TIGMIC_1 (ON811840); 708 (EU697951, EU697952, AF504293); Ganjam G619 (EU697949, EU697950, AF504294); 779 (HM991306, HQ286599, HM991328).

^*b*^ –, no corresponding sequence available in GenBank.

**Fig 1:**
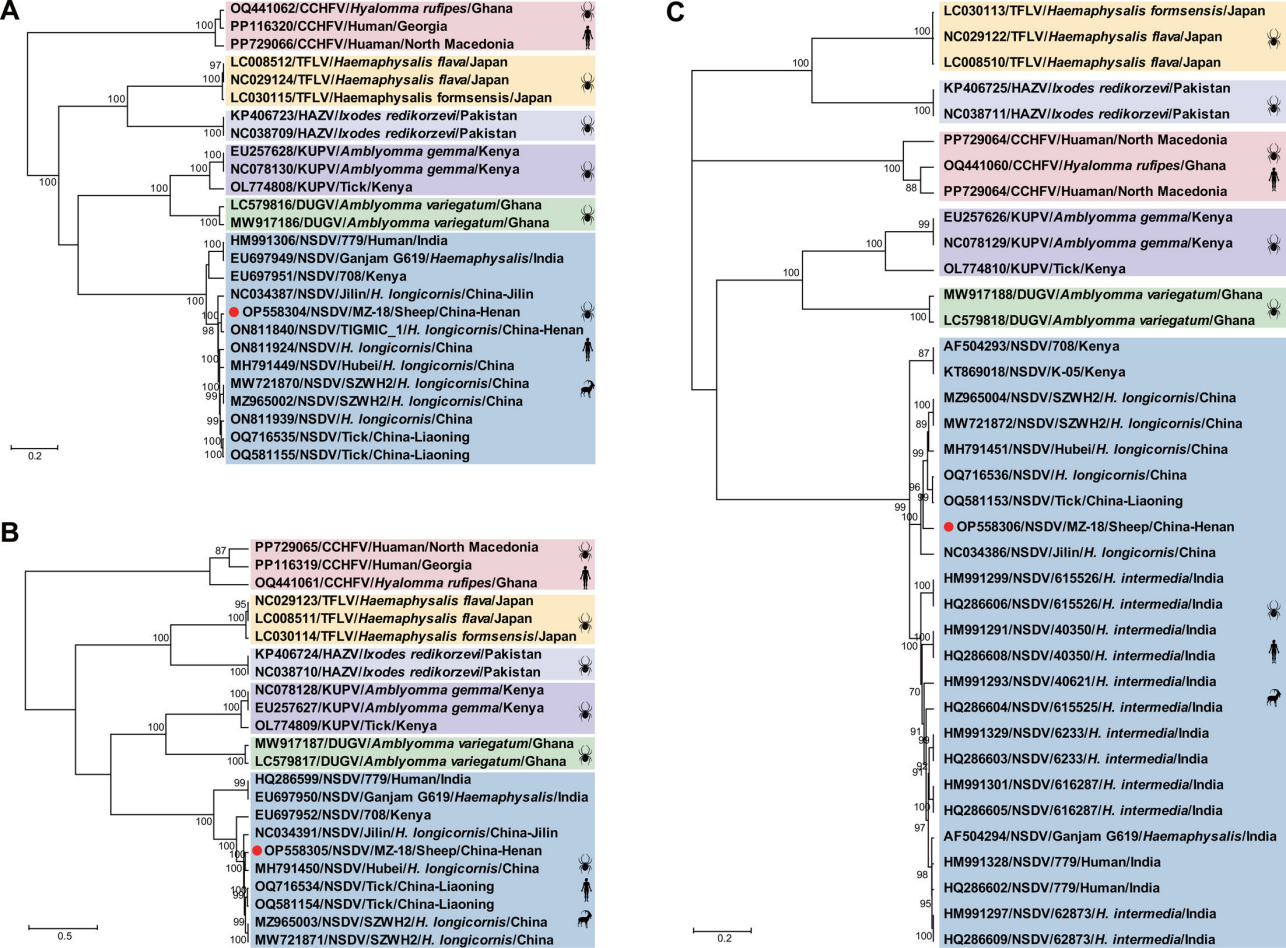
Phylogenetic analysis based on the complete nucleotide sequences of the L (**A**), M (**B**), and S (**C**) segments of NSDV strain MZ-18 and other viruses belonging to the NSD genogroup. The trees were constructed based on the maximum-likelihood method implemented in MEGA 6.0, and mid-point rooted for clarity, and the scale bar represents the number of nucleotide substitutions per site. Bootstrap values were calculated with 1,000 replicates of the alignment, and only bootstrap values >70% are shown at relevant nodes. Sequences obtained in this study are marked with red dots.

Phylogenetic trees reconstructed based on the nucleotide sequence of L, M, and S segments revealed that the NSDV strain MZ-18 formed a monophyletic cluster with other known NSDV strains identified in ticks in China, distinguishing it from NSDV strains identified in Africa and India (Fig. 1). In the M-segment tree, the China-associated NSDV clade showed a closer relationship with the African strain (NSDV/708/Kenya) than with the Indian ones. However, it formed a sister clade to Indian and African viruses in the L- and S-segment trees (Fig. 1). Within the China-associated NSDV clade, the L, M, and S segments of MZ-18 exhibited the closest phylogenetic relationship with NSDV strain TIGMIC_1, Hubei, and Jilin, which were identified in *H. longicornis* from Henan, Hubei, and Jilin provinces of China, respectively (Fig. 1).

### Prevalence and genetic diversity of NSDV in sheep and goats

A total of 679 spleen tissues (569 sheep and 110 goats), including those previously subjected to meta-transcriptomic sequencing, were screened for the presence of NSDV using nested RT-PCR. Of these, 28 individual spleen tissues from sheep and goats tested positive for NSDV, yielding an overall prevalence rate of 4.1% (95% CI: 2.8%–5.9%) (Table 2). The prevalence of NSDV varied significantly among the seven cities surveyed (χ^2^ = 69.723, *P* < 0.001), with rates ranging from 0% to 20.5%. Additionally, the positive rate for NSDV in sheep (4.6%, 95% CI: 3.0%–6.6%) was higher than that in goats (1.8%, 95% CI: 0.2%%–6.4%), although the difference in infection rates between sheep and goats was not statistically significant (χ^2^ = 1.765, *P* = 0.140). In addition, serum antibodies of 539 samples against NSDV-N protein were detected using a competitive enzyme-linked immunosorbent assay (ELISA), revealing that 161 serum samples tested positive, resulting in an overall seroprevalence rate of 29.9% (95% CI: 26.1%–34.0%) (Table 3). The seroprevalence of NSDV infection in sheep and goats ranged from 14.1% (95% CI: 9.5%–19.9%) to 41.3% (95% CI: 33.9%–49.0%). Notably, serum samples collected from Henan Province exhibited the highest rate of NSDV infection.

**TABLE 2:** Prevalence of NSDV in sheep and goats in this study^*a*^

Species	Location							Total (%, 95% CI)
	JZ	NY	LY	SQ	AY	JY	ZZ	
Sheep	8/193	2/10	0/85	0/32	0/71	0/97	16/81	26/569 (4.6, 3.0–6.6)
Goat	0/9	1/70	0/2	0/18	0/9	−	1/2	2/110 (1.8, 0.2–6.4)
Total (%, 95% CI)	8/202 (4.0, 1.7–7.7)	3/80 (3.8, 0.8–10.6)	0/87 (0)	0/50 (0)	0/80 (0)	0/97 (0)	17/83 (20.5, 12.4–30.8)	28/679 (4.1, 2.8–5.9)

^*a*^ JZ, Jiaozuo; NY, Nanyang; LY, Luoyang; SQ, Shangqiu; AY, Anyang; JY, Jiyuan; ZZ, Zhengzhou. NSDV RNA-positive specimens/total specimens; − indicates that no specimens were collected.

**TABLE 3:** Seroprevalence of NSDV in sheep and goats in this study^*a*^

Location/province	Sheep/goat serum samples		
	No. of total samples	No. of positive samples	Positivity rate (%, 95% CI)
Henan Province	172	71	41.3 (33.9–49.0)
Heilongjiang Province	114	45	39.5 (30.5–49.11)
Jilin Province	32	9	28.1 (13.7–46.7)
Ningxia Hui Autonomous Region	191	27	14.1 (9.5–19.9)
Inner Mongolia Autonomous Region	30	9	30.0 (14.7–49.4)
Total	539	161	29.9 (26.1–34.0)

^*a*^ NSDV-positive serum specimens/total specimens. The serum antibody was detected by NSDV-N protein competitive ELISA.

**Fig 2:**
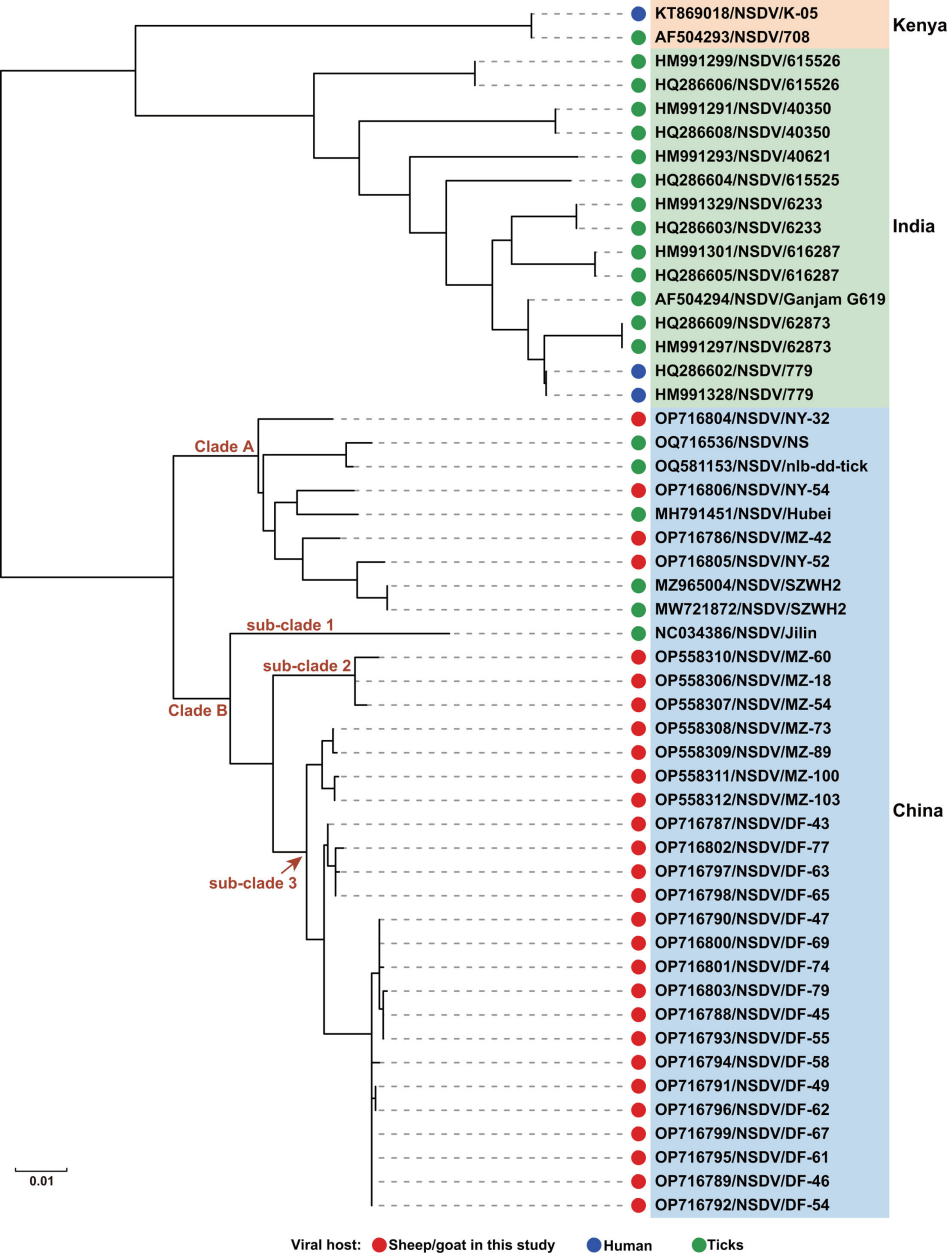
Phylogenetic analysis based on the nearly complete nucleotide sequence of the S segment of NSDV strains identified in this study and known NSDV strains available in GenBank. The trees were constructed based on the maximum-likelihood method implemented in MEGA 6.0, and mid-point rooted for clarity, and the scale bar represents the number of nucleotide substitutions per site. Bootstrap values were calculated with 1,000 replicates of the alignment, and only bootstrap values >70% are shown at relevant nodes. The hosts of NSDV strains discovered are denoted as follows: black dots, ticks; blue dots, human; red dots, sheep or goats in this study.

The nearly complete sequences of the S segment amplified from all positive samples shared 93.7%–100% nucleotide sequence identity with one another. The phylogenetic tree reconstructed based on S segment sequences revealed that all known NSDV strains were clustered into three groups according to their sampling locations: India, Kenya, and China (Fig. 2). Moreover, all NSDV strains identified in China were generally divided into two clades (clade A and B), and clade B was further subdivided into three sub-clades. The NSDV strains identified in this study fell into all two clades and two of the three sub-clades. Notably, NSDV strains identified in ticks and mammalian hosts were clustered together, irrespective of their host species (Fig. 2).

### Isolation, propagation, and host spectrum investigation of NSDV strain MZ-18

**Fig 3:**
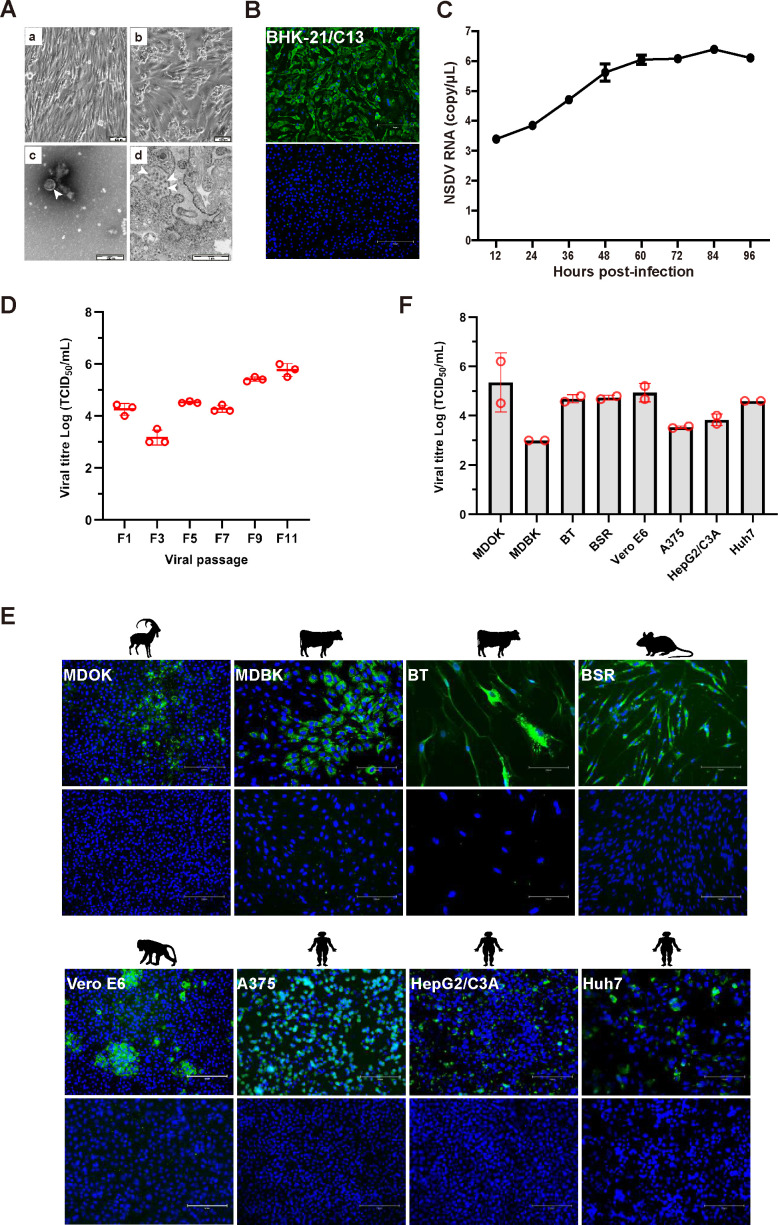
Characterization of NSDV strain MZ-18. (**A**) Isolation of NSDV using BHK-21-C13 cells. The cells were cultured without NSDV infection (**A**) or with NSDV infection post 72 hours (**B**); (**C**) negatively stained NSDV virions (arrows) purified from infected BHK-21-C13 cells; (**D**) transmission electron microscopy showing NSDV virions (arrows) in the cytoplasm of infected BHK-21-C13 cells. (**B**) BHK-21-C13 cells infected with NSDV and fixed at 60 hours post-infection, then incubated with rabbit polyclonal antibodies against NSDV-N protein. Antigens reacting with rabbit IgG are shown in green. (**C**) Growth of NSDV in BHK-21-C13 cells. Supernatants were collected at the indicated time points (12, 24, 36, 48, 60, 72, 84, and 96 hours post-NSDV infection), and viral RNA copies were measured at each time point using quantitative real-time PCR. Mean ± SD (error bars) are shown (*n* = 3). (**D**) Viral titers were determined from BHK-21-C13 cells after serial passaging. Mean ± SD (error bars) are shown (*n* = 3). (**E**) Immunofluorescent staining of NSDV in different cell lines. The cells were infected with NSDV, fixed at 60 hours post-infection, and then incubated with rabbit polyclonal antibodies against the NSDV-N protein. Antigens reacting with rabbit IgG are shown in green. (**F**) Viral titers were determined from the supernatants of different NSDV-infected cell lines. The cells were infected with NSDV strain MZ-18. After 60 hours, the supernatants were collected to infect BHK-21-C13 cells for a 50％ tissue culture infectious dose (TCID_50_) test. Mean ± SD (error bars) are shown (*n* = 2).

After inoculating the supernatant from homogenized positive spleen samples in BHK-21-C13 cells for 72 hours, virus-induced cytopathogenic effects (CPEs) were observed in the infected cells. These effects included cell brightening, shrinkage, clustering, as well as necrosis and dissolution of surrounding cells (Fig. 3A). Additionally, transmission electron microscopy revealed enveloped spherical virions, approximately 80 nm–100 nm in diameter, in both the supernatant and in BHK-21-C13 cells infected with NSDV (Fig. 3A). Immunofluorescent staining with rabbit polyclonal antibodies against the NSDV-N protein demonstrated the presence of viral antigens in the cytoplasm of BHK-21-C13 cells infected with NSDV (Fig. 3B). Furthermore, quantitative real-time RT-PCR (qRT-PCR) targeting the S segment gene, along with the viral titer assessments following serial passaging in BHK-21-C13 cells, confirmed the efficient replication of NSDV within BHK-21-C13 cells (Fig. 3C and D). Notably, the results from the immunofluorescent staining assay and viral titer assessments (Fig. 3E and F) indicated that the NSDV isolated in this study could infect a range of cell lines, including MDOK, MDBK, BT, BSR, Vero E6, A375, HepG2/C3A, and Huh7 cells, suggesting a broad host range and cross-species transmission potential for NSDV.

### Pathogenic characterization of NSDV strain MZ-18

**Fig 4:**
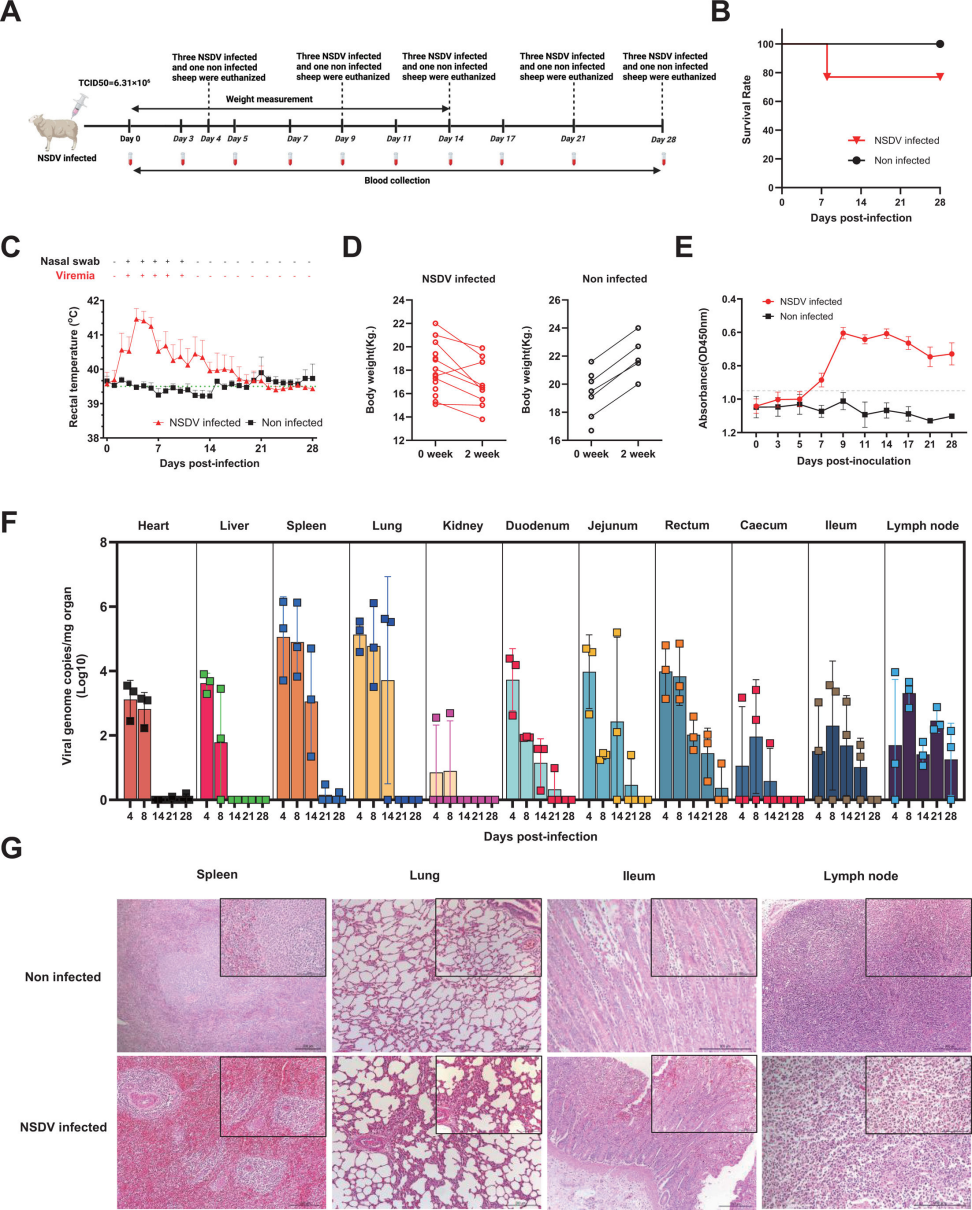
Pathogenic characteristics of NSDV strain MZ-18 in sheep. (**A**) The flow diagram of the animal experiment. The 4-week-old sheep were randomly assigned to one of two groups, either an NSDV infection group (*n* = 15 sheep) or a Dulbecco's modified Eagle medium (DMEM) control group (*n* = 7 sheep). (**B**) Survival rate calculation and survival curve plotting used the Kaplan–Meier method. (**C**) Rectal temperatures of the NSDV-inoculated and mock-inoculated sheep during the 4 weeks of the experiment. Mean ± SD (error bars) temperatures are shown. The viremia and virus excretion in nasal secretions schedule are presented by RT-qPCR assays above the graph. (**D**) Body weight of the NSDV-inoculated (left) and mock-inoculated sheep (right) during the 2 weeks of the experiment. (**E**) Detection of antibodies against NSDV-N protein antigens. The antibody responses (mean OD450 values) detected by NSDV-N protein competitive ELISA in 4-week-old sheep (*n* = 22) following experimental inoculation with NSDV strain MZ-18 or negative control over the course of the infection. Mean ± SD (error bars) of the specific antibodies are shown. (**F**) The viral RNA loads in the organs (heart, liver, spleen, lung, kidney, duodenum, jejunum, rectum, cecum, ileum, and lymph node) of NSDV-inoculated sheep. The organs were collected from sheep which were inoculated with NSDV strain MZ-18, and the viral RNA copies were detected by qRT-PCR. Mean ± SD (error bars) are shown (*n* = 3). (**G**) Histopathological lesions of the spleens, lungs, ilea, and lymph nodes from NSDV- and mock-inoculated sheep. The sheep infected with the NSDV strain MZ-18 showed lymphocyte depletion in white pulp and increased congestion in red pulp of the spleen; mild thickening of alveolar walls and hyperplasia of alveolar epithelial cells in the lung; lamina propria hemorrhage, inflammatory cell infiltration of mucosal epithelium and lamina propria in the ileum and prominent depletion of lymphocytes in the lymph nodes. No obvious pathological lesions were observed in the spleens, lungs, lymph nodes, and ilea of the mock-inoculated sheep.

The design and procedures of the animal experiment are outlined in Fig. 4A, depicting the clinical progression of NSDV strain MZ-18 and mock inoculation in 4-week-old sheep. After an incubation period of 2 to 3 days, the NSDV-infected sheep (15/15, 100%) developed high fever, with peak rectal temperatures of 42.0°C occurring between 4 and 6 days post-infection (dpi) and lasting for 9 to 13 days (Fig. 4C). Diarrhea appeared in all infected sheep at 4–6 dpi, with some showing bloody, watery stools, lasting for 5–6 days and resulting in reduced appetite and poor body condition. Three of the infected sheep (3/12, 25%) died from high fever and severe diarrhea at 8 dpi (Fig. 4B). The remaining infected sheep resumed eating after 2 weeks, gradually improved, and fully recovered by the end of the experiment. Most infected sheep (8/9) experienced significant weight loss during the illness, except for one that showed gained weight, while all non-infected sheep consistently gained weight (Fig. 4D).

Antibody presence against NSDV in the serum of infected sheep was evaluated using a competitive ELISA targeting the NSDV-N protein, with seroconversion evident starting at 7 dpi (Fig. 4E). qRT-PCR analysis revealed detectable NSDV viral genomes in various tissues including the heart, liver, spleen, lung, kidney, duodenum, jejunum, rectum, cecum, ileum, and lymph node of infected sheep at both 4 dpi and 8 dpi (Fig. 4F). Notably, viral RNA loads in the spleen and lung were higher than in other tissues at 4 dpi, 8 dpi, and 14 dpi (Fig. 4F). Maximum viral level was detected in the spleen of one sheep at 4 dpi with 10^6.151^ copies/m. By 28 dpi, all tested organs were NSDV-negative, except lymphoid tissues, which showed low viral RNA loads (Fig. 4F). These results indicate that NSDV replicates more efficiently in the spleen and lung, with illness caused by NSDV strain MZ-18 lasting 1 to 2 weeks before complete recovery.

Histopathological examinations of spleens, lungs, ilea, and lymph nodes from NSDV-infected sheep revealed notable pathological changes: lymphocyte depletion in white pulp and increased congestion in red pulp of the spleen; mild thickening of alveolar walls and alveolar epithelial cell hyperplasia in the lungs; lamina propria hemorrhage and inflammatory cell infiltration in the ileum; and significant lymphocyte depletion in lymph nodes (Fig. 4G). In contrast, mock-infected sheep showed no significant pathological changes.

## DISCUSSION

NSDV is a highly lethal pathogen that causes significant economic losses in sheep and goats. It was historically thought to be endemic mainly to Africa and South Asia until it was detected in China (9, 10). Although viral RNA has been detected in *H. longicornis* ticks across various regions of China (18–23), there have been no prior reports of NSDV infection in sheep or goats in China. Our findings derived from meta-transcriptomics sequencing, molecular and seroprevalence epidemiological investigations, virus isolation, basic biological characterization, and animal experiments provide compelling evidence of NSDV infection in sheep and goats in China. This study marks the first identification of NSDV in sheep and goats within the country and expands the known geographic distribution of NSDV globally.

NSDV is primarily transmitted through the feeding of competent infected ticks, resulting in a geographic distribution closely associated with the presence of tick vectors (9). In Africa, *Rhipicephalus appendiculatus* ticks are the primary carriers of NSDV (24), while *Haemaphysalis intermedia* ticks are involved in South Asia (10, 12). In China, NSDV has been found in *H. longicornis* ticks from various regions (18–23). The NSDV strains identified in this study showed the highest similarity to those found in *H. longicornis* ticks in China, suggesting that *H. longicornis* ticks may serve as the main vector for NSDV transmission in East Asia. Given that *H. longicornis* ticks are widespread and dominant in China (25), this suggests a potentially broader distribution of NSDV within the country. Additionally, the seroprevalence investigations conducted in this study further confirm the presence of NSDV infection among sheep and goats in China. Therefore, extensive surveys and monitoring of NSDV in sheep, goats, and different tick species are crucial for understanding its prevalence across China.

A previous study revealed a strong geographical structure in the phylogeny of NSDV, closely aligned with sampling locations such as India, Kenya, and China (20). Consistent with this, the NSDV strains identified in this study clustered with strains previously found in China. Furthermore, NSDV strains in China were categorized into various clades and sub-clades, indicating the local circulation of NSDV among ticks and vertebrate hosts within the country. Notably, NSDV strains identified in both ticks and mammals in China were closely related phylogenetically, similar to the relationships observed between strains from India and Kenya. Additionally, NSDV proteins, especially the glycoproteins Gn and Gc, were highly conserved among strains from ticks and mammals, implying that significant variations in genome structure are not necessary for NSDV transmission between ticks and mammals.

Although the viral RNA of NSDV has been detected in *H. longicornis* ticks across a broad geographical range in China (18–23), no clinical cases of NSDV infection in sheep or goats have been reported in this country. In this study, we isolated NSDV from sheep and goats for the first time in China, strongly suggesting that NSDV infections are present in these livestock populations and highlighting the urgent need to further investigate NSDV epidemiology in China. Notably, previous studies have provided serological evidence of NSDV infection in humans (13–16). Our study demonstrates that the NSDV strain MZ-18 can replicate in various cell lines, including human cell lines (A375, HepG2/C3A, and Huh7), suggesting the cross-species transmission potential of the strain isolated in China.

This study explores the pathogenesis of NSDV strain MZ-18 infections in sheep, offering new insights into the clinical progression, immune response, and pathological effects of the infection. Clinical signs such as high fever emerging 2 to 3 days post-infection and diarrhea developing between 4 and 6-days post-infection align with established clinical presentations of NSDV in sheep and other small ruminants (26, 27). The fever persisted for 9 to 13 days, compounded by diarrhea that sometimes led to bloody stools, consistent with previously documented clinical signs. Although NSDV is recognized for being highly pathogenic for small ruminants, reported mortality rates vary significantly. In our study, the mortality rate among infected sheep was 25%, with three deaths attributed to severe clinical signs, including high fever and diarrhea. Notably, the majority of sheep were gradually improved and fully recovered by the end of the experiment. This contrasts with previous reports of mortality as high as 90% (10), which may stem from differences in virus strains, infectious doses, modes of infection, and sheep breeds, underscoring the necessity for further research to clarify these factors.

This study had several limitations. First, NSDV was discovered occasionally during the investigation of spleen virome through high-throughput meta-transcriptomic sequencing, thus we could not trace the clinical and pathological manifestation of sheep or goats infected with NSDV in nature. Additionally, all the spleen and serum samples were collected from certain regions, which represent a limited survey to reveal the entire epidemiological characteristics of NSDV in China. Nevertheless, our study provided the first evidence of NSDV infection in sheep and goats in China and emphasized the importance of NSDV surveillance to prevent potential outbreaks of NSD in China.

## MATERIALS AND METHODS

### Sample collection and total RNA extraction

In a pilot viral agent discovery project, a total of 90 spleen tissues from sheep and goats were collected at a local slaughterhouse located in Mengzhou city, Henan province, China, during June 2021 to January 2022. Approximately 50 mg of spleen tissue was homogenized with 500 µL sterile phosphate-buffered saline (PBS), and total RNA was extracted from 200 µL of the homogenates using TRIzol LS reagent (Invitrogen, Carlsbad, CA, USA) and purified according to the manufacturer’s instructions. The quantity and quality of the extracted RNA were evaluated with a NanoDrop 2000 (Thermo Fisher Scientific, Waltham, MA, USA). Subsequently, 30 individual RNA samples were mixed as one pool in equal volumes. These pooled RNA samples were then subjected to meta-transcriptomic sequencing. The quality of the pooled RNA was assessed using an Agilent 2100 Bioanalyzer (Agilent Technologies*,* Santa Clara, CA, USA) prior to library construction and sequencing.

### Virus discovery and viral abundance estimation

RNA library preparation was conducted following the previously described methodology (28). Briefly, ribosomal RNA (rRNA) was depleted using a Ribo-Zero-Gold (Epidemiology) kit (Illumina Inc., San Diego, CA, USA). The remaining RNA was then fragmented, reverse-transcribed, adapted, and purified using the TruSeq total RNA library preparation kit (Illumina Inc.). Library quality was assessed using the Qubit (Thermo Fisher Scientific, USA) high-sensitivity RNA/DNA assays and the Agilent 2100 Bioanalyzer (Agilent Technologies, USA). Paired-end sequencing (150 bp) was performed on the Illumina Novaseq platform. All library preparation and sequencing procedures were conducted by Novogene (Tianjin, China).

Sequencing reads (raw data) were demultiplexed, adapter-trimmed, and quality-controlled to generate the clean reads with the fastp program (29). *De novo* assembly was performed using the Megahit program with default settings (30), and the resulting contigs were compared to the non-redundant protein (nr) database using the Diamond Blastx program (31) with an e-value threshold at 1E − 4. Viral contigs were identified based on taxonomy information from the BLAST hit results. Subsequently, viral contigs with unassembled overlaps were merged using the SeqMan program implemented in the Lasergene software package (version 7.1, DNAstar, USA). To confirm the assembly results, reads were mapped back to the target contigs with Bowtie2 (32), and assembly errors were inspected and corrected using the Integrated Genomics Viewer (33). Gaps between these contigs were filled by RT-PCR and Sanger sequencing. The genome termini of the virus were determined using RACE kits (code no. 634858, TaKaRa, Dalian, China) following the manufacturer’s instructions. The final virus genome sequences were obtained for the majority consensus of the mapping assembly and confirmed by Sanger sequencing with overlapping primers that covered the entire sequence. The primers used in sequence conformation and extension are listed in Table S1.

To estimate the abundance of NSDV in the library, sequencing reads associated with rRNA were removed by mapping to the SILVA database (https://www.arb-silva.de/) using Bowtie2 (32). The remaining reads were subsequently mapped to the confirmed viral genomes using the “end-to-end” setting, and the relative viral abundance was estimated using the percentage of non-rRNA reads that were mapped to the target genome and normalized as RPM.

### Genomic characterization

The potential open reading frames (ORFs) were predicted using ORFfinder and subsequently compared with sequences from other NSD genogroup viruses. To analyze the GPC domains and post-translational modification features, several tools were independently utilized to predict specific structural features: TMHM for transmembrane protein prediction (34), SignalP 4.0 for predicting signal peptidase cleavage sites (35), NetNGlyc−1.0 for predicting N-linked glycosylation sites (36), and NetOGlyc for predicting mucin-type O-glycosylation sites (37). The protein sequence was individually input into the respective tools, and all analyses were conducted using the default parameters recommended by the tool developers to ensure reproducibility and optimal prediction accuracy. Additionally, conserved domains or motifs within the NSDV genome were identified by comparing the sequences in the NCBI Conserved Domain Database (https://www.ncbi.nlm.nih.gov/Structure/cdd/cdd.shtml) and referencing conserved structures previously found in other NSD genogroup viruses (38–43).

### Molecular screening of NSDV in sheep and goats

To gain insight into the prevalence and genetic diversity of NSDV infecting sheep and goats, spleen tissues were collected from small local slaughterhouses in seven cities of Henan (Fig. S7). Total RNA extracted from individual spleen samples was subjected to NSDV screening using the primers NSDV-fwd, NSDV-rev1, and NSDV-rev2, which target the coding region of the L segment (listed in Table S1). The nested PCR was performed with the PrimeScript One Step RT-PCR kit (TaKaRa) according to the manufacturer’s instructions. Briefly, the reverse transcription was carried out at 42°C for 30 min, followed by the first round of PCR amplification using the primer pair NSDV-fwd and NSDV-rev1 at an annealing temperature of 53°C for 30 seconds per cycle. For the second round of PCR, a PCR mix containing Taq DNA polymerase was used, with the primer pair NSDV-fwd and NSDV-rev2 annealing temperature also set at 53°C. The PCR products of expected size were confirmed by Sanger sequencing. Nearly complete sequences of the S segment were obtained from NSDV positive samples to investigate the genetic diversity of NSDV identified in this study. The sequence of all primer pairs used in this study is listed in Table S1.

### Seroprevalence investigation of NSDV infection

To confirm the presence of NSDV infection in sheep and goats in China, 539 serum samples were collected from the provinces or Autonomous Regions including Henan, Heilongjiang, Jilin, Ningxia, and Inner Mongolia (Table 3). Antibodies against NSDV were subsequently detected using a competitive ELISA based on NSDV-N protein that we developed. The optical density (OD) was measured at 450 nm using an ELISA plate reader (PE, USA). Serum sample with an OD450 value equal to or less than the cut-off was classified as NSDV antibody positive.

### Sequence comparison and phylogenetic analysis

The nucleotide and amino acid identities of each segment of NSDV were calculated using the ClustalW method implemented in the MegAlign program available in the Lasergene software package (version 7.1, DNAstar). To infer the phylogenetic relationship between the NSDV strain identified in this study and other known NSD genogroups of orthonairoviruses, the maximum likelihood method phylogenetic trees based on the nucleotide sequences of L, M, and S segments were reconstructed in MEGA 6.0 software employing the general time-reversible (GTR) nucleotide substitution model and the optimized parameters of gamma (*Γ*)-distribution and proportion of invariable sites (i.e., GTR + *Γ* + I) with bootstrap support values calculated from 1,000 replicates. All phylogenetic trees were mid-point rooted for purposes of clarity only.

### Virus isolation and transmission electron microscopy observation

According to the recommendation of the WOAH, the baby hamster kidney clone 13 cell line was employed for virus isolation (44). In brief, cells were cultured in minimum essential medium (MEM, Gibco) supplemented with 10% fetal bovine serum (Gibco) and 1% penicillin-streptomycin in 5% CO_2_ at 37°C. Positive spleen samples were homogenized and centrifuged at 12,000 × *g* for 10 min at 4°C. The resulting supernatant was filtered through a 0.22 µm filter (Millipore, USA), diluted 1:10 with serum-free MEM, and then inoculated onto a confluent monolayer of BHK-21-C13 cells. Following a 2 hour incubation at 37°C with 5% CO_2_, the inoculum was removed, the cells were washed twice with PBS, and further cultured in MEM supplemented with 2% fetal bovine serum (FBS) for 96 hours. Virus-induced CPEs were monitored daily, and virus isolates were confirmed by RT-PCR.

The NSDV-infected BHK-21-C13 cells and supernatants were harvested 96 hours post-inoculation. The cells underwent two washes with PBS and were centrifuged at 1,000 × *g* for 5 min. Subsequently, the cell pellets were fixed overnight at 4°C with 2.5% (wt/vol) glutaraldehyde and post-fixed for 1 hour at room temperature with 1% OsO_4_ in cacodylate buffer. The pellets were then dehydrated in ethanol and rinsed with propylene oxide for 30 min at room temperature. After embedding in resin, the samples were examined using a transmission electron microscope (H-7650, Hitachi High-Tech). The supernatant underwent similar treatment and observation via transmission electron microscopy as described previously (45).

### Virus propagation and host spectrum investigation

To evaluate the propagation of NSDV in BHK-21-C13 cells, quantitative real-time RT-PCR was employed to target the S segment gene (NSDV-S-F: TGCAGAAAGCCCTTGAACTA, NSDV-S-R: TGAGACTGTC GGGAACATCT, NSDV-S-Prob: FAM-AGTCACACCTGCCTTCCAAAGCCAGTA G-BHQ1). Supernatants from NSDV-infected BHK-21-C13 cells were harvested at 12, 24, 36, 48, 60, 72, 84, and 96 hours post-infection. Quantitative real-time PCR was conducted using the TaKaRa One Step PrimeScript RT-PCR kit. The viral copy numbers were determined by correlating the Ct values with a standard curve established using *in vitro* transcribed RNA containing the targeted S segment sequence. Additionally, the supernatants from passage 1, 3, 5, 7, 9, and 11 of NSDV-infected BHK-21-C13 cells were collected, and the viral titer for each passage was assessed.

To investigate the host spectrum of NSDV isolated in this study, we infected MDOK (ATCC, CRL-1633), MDBK (ATCC, CCL-22), BT (ATCC, CRL-1390), BSR (CVCL_RW96), Vero E6 (ATCC, CRL-1586), A375 (ATCC, CRL-1619), HepG2/C3A (ATCC, HB-8065), and Huh7 (JCRB0403) cell lines with NSDV at a multiplicity of infection (MOI) = 1 or left uninfected, respectively. After 60 hours of infection, virus detection was performed using the immunofluorescent staining as described below.

### Immunofluorescent staining of NSDV-infected cells

Cells infected with NSDV were fixed with 10% formalin for 30 min at 60 hours post-infection and then rinsed twice with PBS. After permeabilization with 0.1% Triton X-100 in PBS for 5 min, the cells were incubated with rabbit polyclonal antibodies against the NSDV-N protein, diluted 1:250 in PBS. The cells were washed three times with PBS. Subsequently, the antigens were visualized using goat anti-rabbit IgG (H + L) conjugated with Alexa Fluor 488 (Thermo Fisher Scientific), diluted 1:1,000 in PBS. Finally, counterstaining was performed using 4′,6-diamidino-2-phenylindole (DAPI), followed by three washes with PBS.

### Animal challenge

A total of 22 four-week-old sheep, free of brucella, *Escherichia coli* K99, bovine rotavirus, and coronavirus (confirmed by real-time PCR), were sourced from Hengtai Lake Sheep Breeding Base in Baiquan county, Qiqihar City, Heilongjiang Province, China. The sheep were randomly divided into two groups: 15 for NSDV inoculation and 7 for mock infection, and housed separately under Biosafety Level 2 conditions. The inoculated group received a subcutaneous challenge in the neck with the NSDV strain MZ-18 at 6.31 × 10^6^ TCID_50_, while the control group received DMEM. Sheep were monitored daily for clinical signs and rectal temperatures, and blood samples were collected at 0, 3, 5, 7, 9, 11, 14, 17, 21, and 28 dpi for NSDV and antibody detection using the competitive ELISA based on the NSDV-N protein. Three infected sheep and one control sheep were humanely euthanized at 4, 9, 14, 21, and 28 dpi, respectively. Tissue sections (heart, liver, spleen, lung, kidney, duodenum, jejunum, rectum, cecum, ileum, and lymph node) were fixed in 10% phosphate buffered formalin for histological analysis or stored at −70°C for virus quantitation.

### Viral RNA load and histopathology examination

Total RNA was isolated from each tissue using the EasyPure Viral DNA/RNA Kit (TransGen Biotech Co.) following the manufacturer’s instructions. TaqMan fluorescent qRT-PCR was conducted to quantify the NSDV viral RNA loads in tissues collected at 4, 9, 14, 21, and 28 dpi. The sequences of the primers used are as described above. Tissue samples were fixed in 10% phosphate buffered formalin, embedded in paraffin, cut into 4 µm sections, and stained with hematoxylin and eosin (H&E) according to the standard procedure. The samples were visualized using an Olympus BX43 bright-field microscope (Olympus Corporation, Tokyo, Japan).

## Data Availability

All sequences generated in this study have been submitted to GenBank under accession numbers OP558304−OP558312 and OP716786−OP716806. The data sets used and/or analyzed during the current study are available from the corresponding author on reasonable request.

## SUPPLEMENTAL MATERIAL

Supplemental material jvi.00006-25-s0001.pdf Figures S1 to S7 and Table S1.

ASM does not own the copyrights to Supplemental Material that may be linked to, or accessed through, an article. The authors have granted ASM a non-exclusive, world-wide license to publish the Supplemental Material files. Please contact the corresponding author directly for reuse.
