# Supplementary material for: Epidemiological, virological, and pathogenic insights into Nairobi sheep disease virus infection in sheep and goats in China
Source: J Virol. 2025 May 21;99(6):e00006-25. doi: 10.1128/jvi.00006-25 (PMC12172417; doi:10.1128/jvi.00006-25)
Supplement: Supplemental material — Figures S1 to S7 and Table S1. [file jvi.00006-25-s0001.pdf]

Epidemiological, virological, and pathogenic insights into Nairobi sheep disease virus infection in sheep and goats in China

Supplementary Material

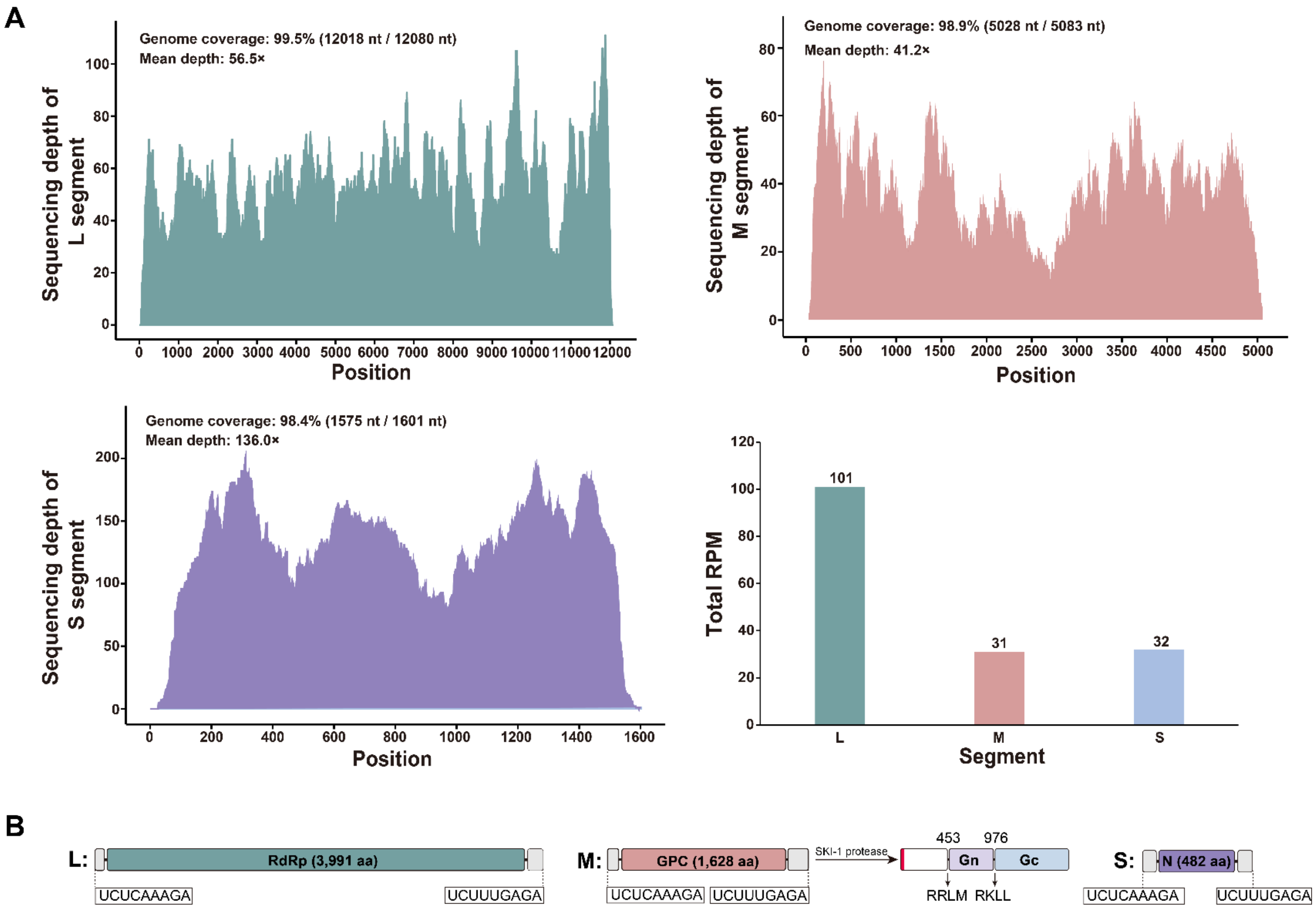

Fig. S1. Meta-transcriptomics sequencing analysis and genetic characterization of NSDV strain MZ-18. **(A)** Sequencing depth and relative viral abundance estimation of NSDV strain MZ-18. Non-rRNA reads were mapped and counted on the L, M, and S segments of the NSDV strain MZ-18 genome, with histograms showing depth at each base position. Relative viral abundance was estimated as the RPM of the L, M, and S segments of NSDV strain MZ-18. **(B)** Schematic diagram of NSDV strain MZ-18 genomic RNA segments. The viral genome consists of large (L), medium (M), and small (S) segments RNA encoding the predicted large protein (L), glycoprotein precursor (GPC), and nucleoprotein (N), respectively. The SKI-1 protease cleavage sites and sequences of GPC in the M segment are indicated with black arrows.

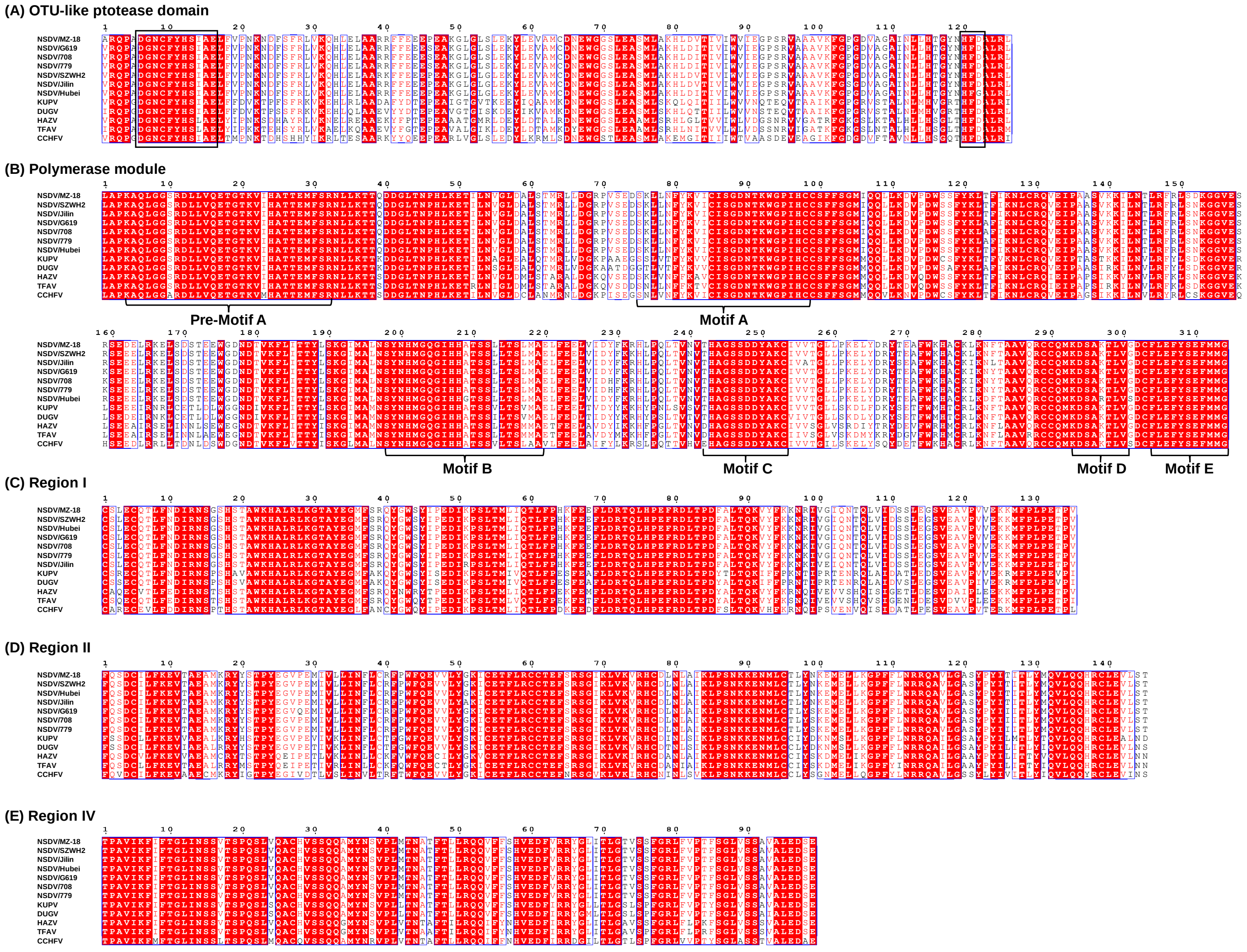

Fig. S2. Amino acid sequence alignment of the RdRp conserved regions of of NSDV and other NSD genogroup viruses. Conserved bases are shaded red. **(A)** Ovarian tumor domain (OTU)-like cysteine proteases motif, conserved residues are boxed; **(B)** Polymerase module, pre-Motif A and Motif A–E are underlined; **(C)** Region I; **(D)** Region II; **(E)** Region IV are highly conserved in bunya- and arenaviruses. NSDV, Nairobi sheep disease virus; KUPV, Kupe virus; DUGV, Dugbe virus; HAZV, Hazara virus; TFLV, Tofla virus; CCHFV, Crimean-Congo hemorrhagic fever virus.

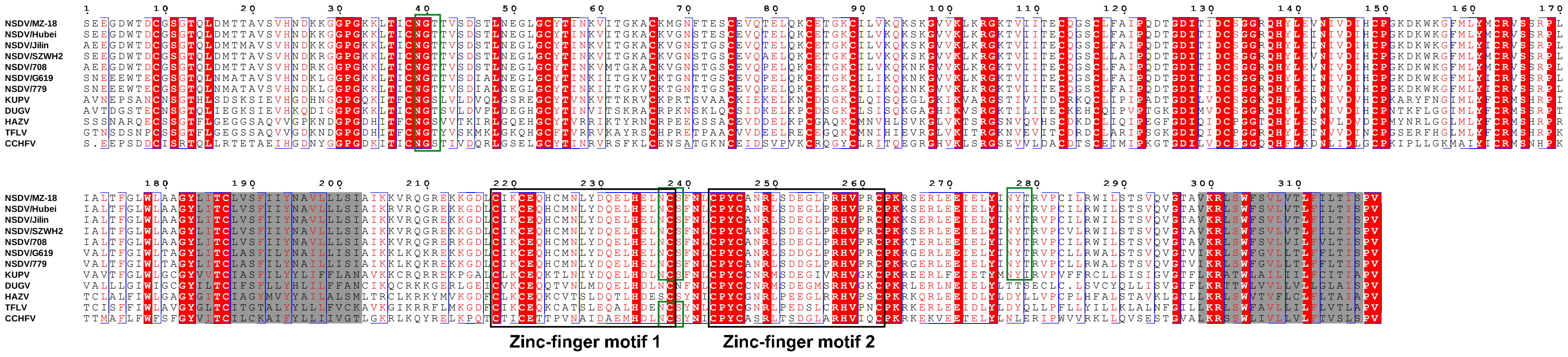

Fig. S3. Amino acid sequence alignment of the Gns of NSDV and other NSD genogroup viruses. Conserved bases are shaded red. Two transmembrane domains are shaded gray and two C-terminus localized zinc-finger motifs are boxed with black line. Putative *N*-glycosylation sites are boxed with green. NSDV, Nairobi sheep disease virus; KUPV, Kupe virus; DUGV, Dugbe virus; HAZV, Hazara virus; TFLV, Tofla virus; CCHFV, Crimean-Congo hemorrhagic fever virus.

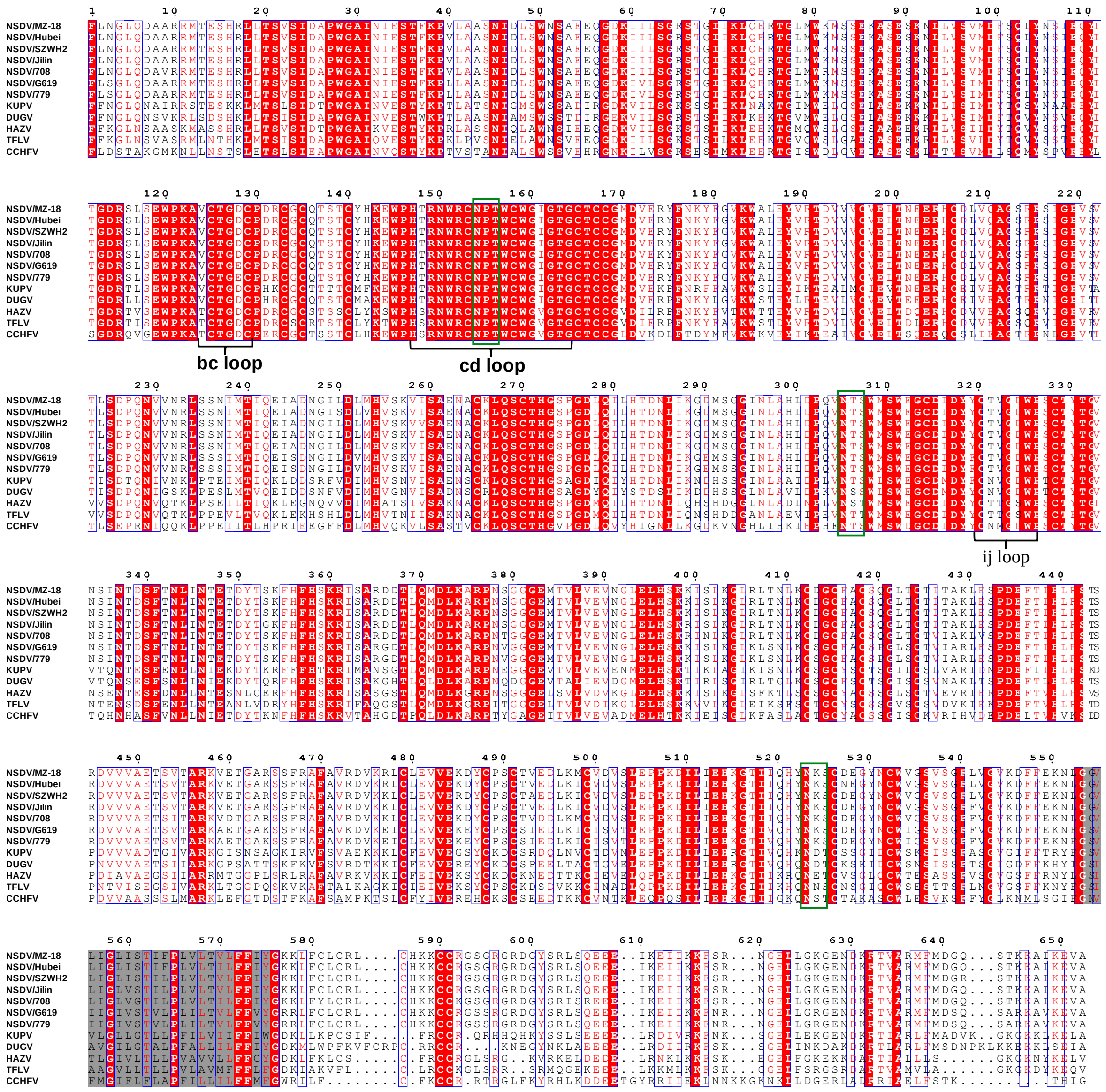

Fig. S4. Amino acid sequence alignment of the Gcs of NSDV and other NSD genogroup viruses. Conserved bases are shaded red. Transmembrane domain is shaded gray and putative *N*-glycosylation sites are boxed with green. Fusion loops are underlined. NSDV, Nairobi sheep disease virus; KUPV, Kupe virus; DUGV, Dugbe virus; HAZV, Hazara virus; TFLV, Tofla virus; CCHFV, Crimean-Congo hemorrhagic fever virus.

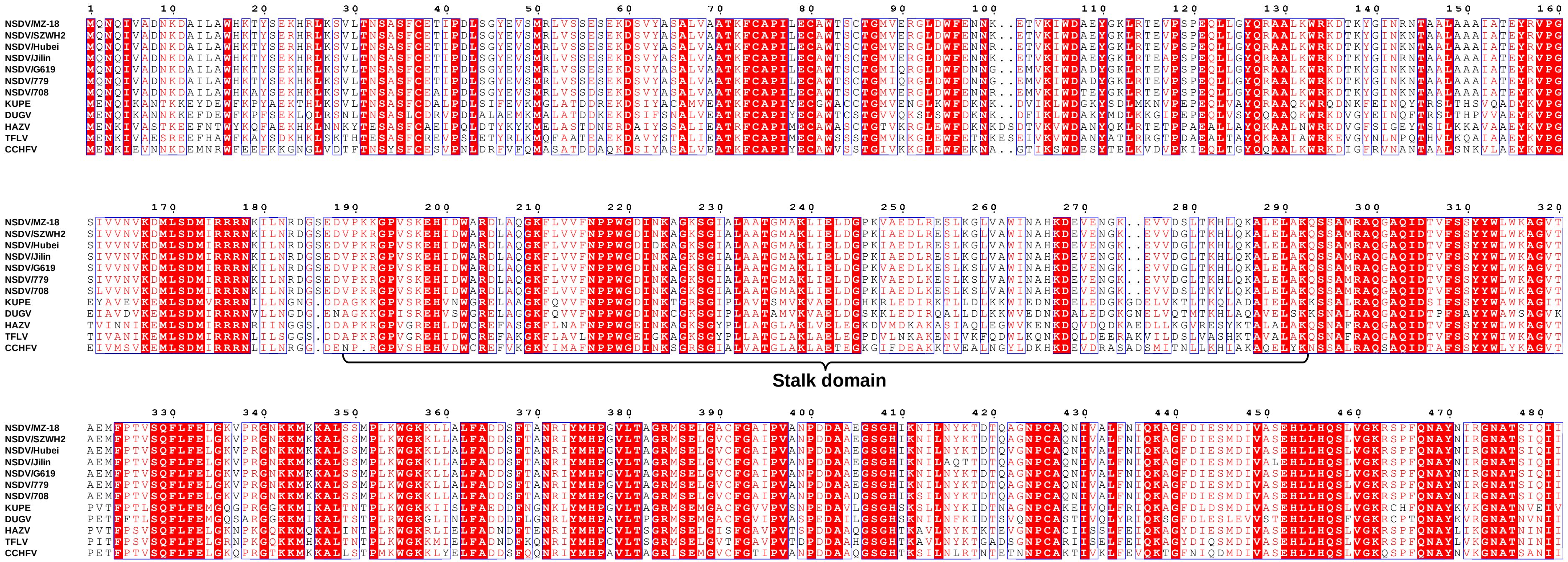

Fig. S5. Amino acid sequence alignment of the nucleoproteins of NSDV and other NSD genogroup viruses. Conserved bases are shaded red. The flexible stalk domain is underlined. NSDV, Nairobi sheep disease virus; KUPV, Kupe virus; DUGV, Dugbe virus; HAZV, Hazara virus; TFLV, Tofla virus; CCHFV, Crimean-Congo hemorrhagic fever virus.

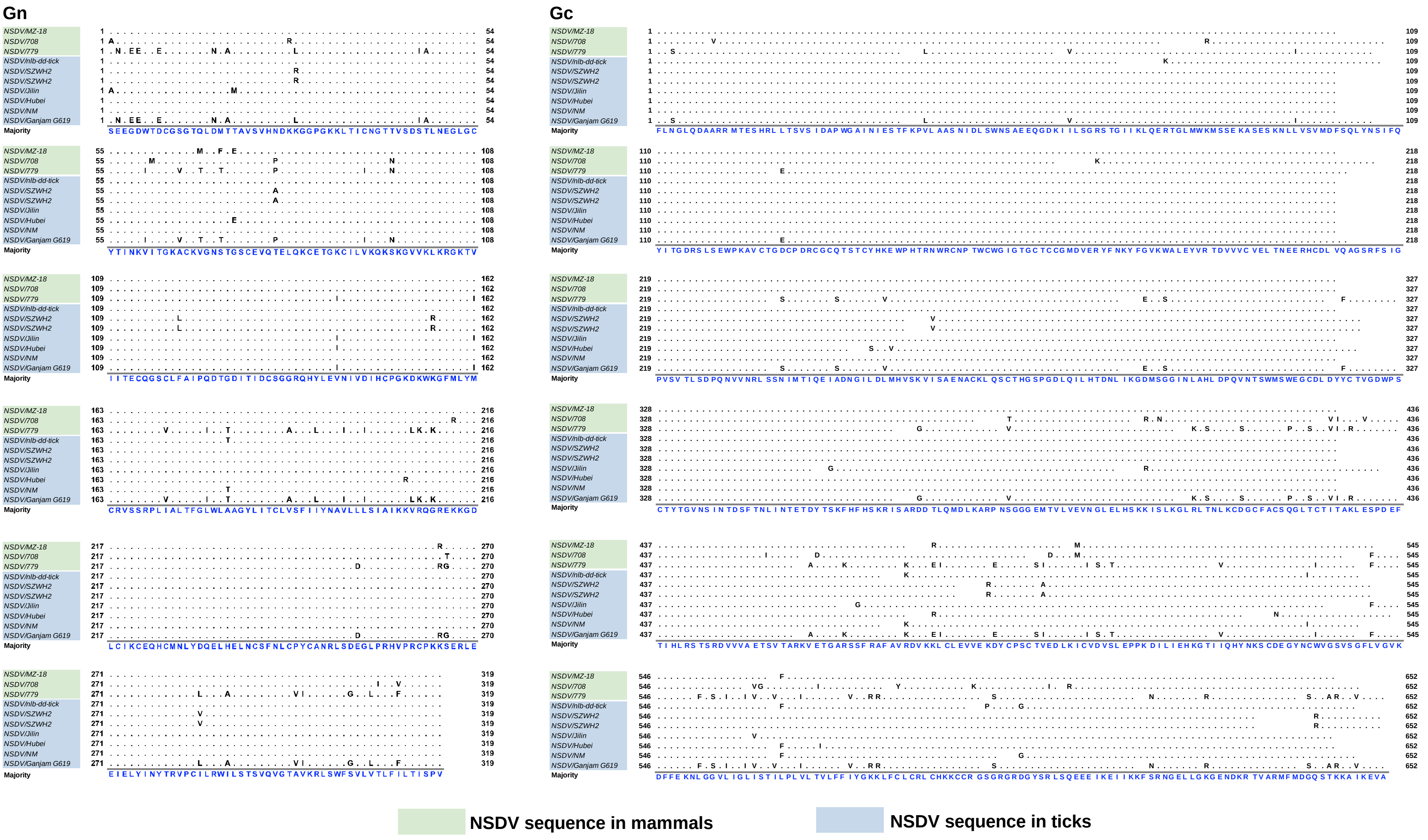

Fig. S6. Multiple sequence alignment of Gc and Gn proteins of NSDV strains currently available from GenBank. The amino acid sequences were aligned using the Clustal W algorithm and visualized using the Jalview software (version 2.11.1.4).

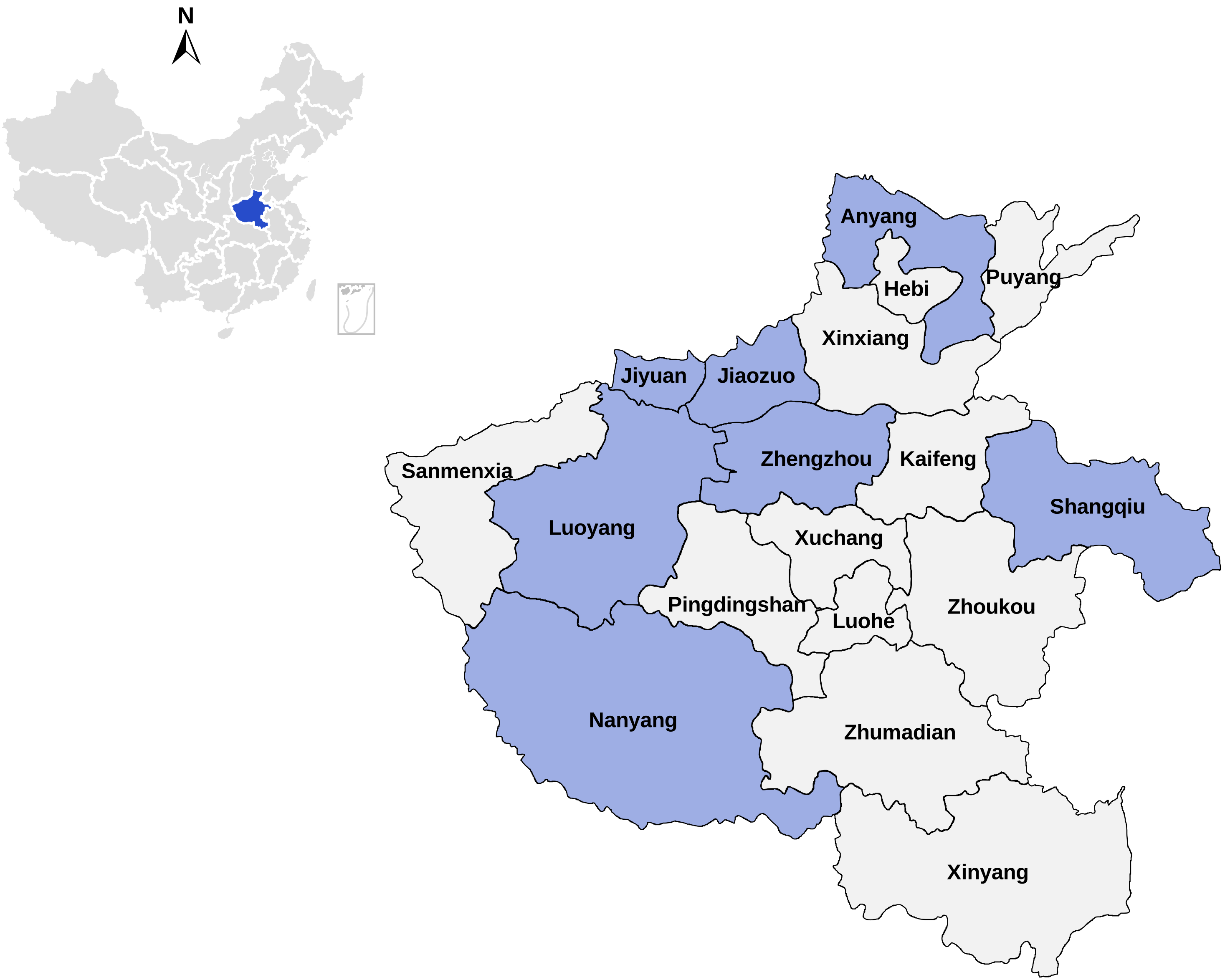

Fig. S7. Geographic maps showing the location of sampling sites where the spleen tissues were collected in this study. This map was plotted by combination of Surfer software version-4 (Golden Software, USA) and Adobe illustrator version CC2017 (Adobe, USA).

Table S1. Primer sequences used in this study.

Primer name	Sequence	Amplification
NSDV-fwd	GCAGAAGGACTCCAAGTGTTC	Molecular screening of NSDV
NSDV-rev1	TCTAATGACAGGCTGGCTATCA	
NSDV-rev2	CACCTGTGCTGTTGTAAGACT	
L-150F	CCTGGAGATGTTGCTGGTG	Sequence confirmation of L segment
L-183F	CACACTGGCTACAATCACTT	
L-1715R	GCTTGGCTTAATGTCTTCTGG	
L-1540F	AAGAGTTGCCTGAGGAATGTT	
L-1593F	AGGAACTCTGGCAGTCACA	
L-3129R	GCAGCCTGTTCATTATGTTAGT	
L-3047F	CGCTATCCTTAGGTCACACT	
L-4780R	TCAGCAGTCTCTTCTAATACAC	
L-4666R	ACACCATAGACGGAGAAACAT	
L-4380F	GGACTCATCATAAGAGACAACC	
L-6155R	GTGACGGACCAAGTAAGACT	
L-5934R	ACAGTTCTCCTGCCATCAC	
L-5784F	AAGGAACTGACTGGAAGCAT	
L-7316R	TGATGAGGTGGCATGATGTAT	
L-7340R	TTCTGCCATTAGCGAGGTC	
L-7184F	GAGTGACAGCACCGAAGAG	
L-8872R	GTATCCAGCCAGTGAGTGAAT	
L-8742R	AGTACGACTTGAGGAAGATGG	
L-8606F	CGTCGCCACAGAAGAGTA	
L-10138R	TCGCTTAGTGTAGGTAGGAAC	
L-10109R	TTGCTGGTGTTGCTCCTT	
L-10013F	GGAGTCAACTGGTCTACTATGT	
L-10018F	CAACTGGTCTACTATGTGAGGT	
L-11650R	CTGGATGATTCTTCGCTAGGT	
M-113F	AAGGCAGAAGATGAACAAGAC	Sequence confirmation of M segment
M-158F	CCAGACCAGCCAGAACAA	
M-1764R	ACAGCGTTATAGATGATGAAGG	
M-1525F	CCTCTTTGCCATCCCACAG	
M-1539F	CACAGGACACAGGAGACATC	
M-3039R	TCCATCACTGAAACAAGAAGGT	
M-2932F	TGGAAGGTCAACAGGAATCATA	
M-2965F	AAGAACTGGTCTGATGTGGAA	
M-4526R	CTGAGCCTCTACAACACTTCT	
S-20F	GCTTTCGCCCACATTGTCT	Sequence confirmation of S segment
S-26F	GCCCACATTGTCTTTGAACTT	
S-1539	AGAAGCGAGCAGCAGGAA	
